# Antegrade Intramedullary Femoral Lengthening and Distal Temporary Hemiepiphysiodesis for Combined Correction of Leg Length Discrepancy and Coronal Angular Deformity in Skeletally Immature Patients

**DOI:** 10.3390/jcm12083022

**Published:** 2023-04-21

**Authors:** Andrea Laufer, Adrien Frommer, Georg Gosheger, Gregor Toporowski, Jan Duedal Rölfing, Carina Antfang, Robert Roedl, Bjoern Vogt

**Affiliations:** 1 Pediatric Orthopedics, Deformity Reconstruction and Foot Surgery, Muenster University Hospital, 48149 Muenster, Germany; adrien.frommer@ukmuenster.de (A.F.); gregor.toporowski@ukmuenster.de (G.T.); jan.rolfing@rm.dk (J.D.R.); carina.antfang@ukmuenster.de (C.A.); roedlr@ukmuenster.de (R.R.); bjoern.vogt@ukmuenster.de (B.V.); 2 General Orthopedics and Tumor Orthopedics, Muenster University Hospital, 48149 Muenster, Germany; georg.gosheger@ukmuenster.de; 3 Children’s Orthopedics and Reconstruction, Aarhus University Hospital, 8200 Aarhus, Denmark

**Keywords:** leg length discrepancy, angular deformity, distraction osteogenesis, temporary hemiepiphysiodesis, PRECICE^®^ lengthening nail, FlexTack^TM^ guided growth staple

## Abstract

Leg length discrepancies (LLD) are frequently associated with coronal malalignment. Temporary hemiepiphysiodesis (HED) is a well-established procedure for the correction of limb malalignment in skeletally immature patients. For treatment of LLD > 2 cm, lengthening with intramedullary devices gains increasing popularity. However, no studies have investigated the combined application of HED and intramedullary lengthening in skeletally immature patients. This retrospective single-center study evaluated the clinical and radiological outcomes of femoral lengthening with an antegrade intramedullary lengthening nail combined with temporary HED performed in 25 patients (14 females) between 2014 and 2019. Temporary HED through the implantation of flexible staples of the distal femur and/or proximal tibia was either performed prior (*n* = 11), simultaneously (*n* = 10) or subsequently (*n* = 4) to femoral lengthening. The mean follow-up period was 3.7 years (±1.4). The median initial LLD was 39.0 mm (35.0–45.0). Twenty-one patients (84%) presented valgus and four (16%) showed varus malalignment. Leg length equalization was achieved in 13 of the skeletally mature patients (62%). The median LLD of the eight patients with residual LLD > 10 mm at skeletal maturity was 15.5 mm (12.8–21.8). Limb realignment was observed in nine of seventeen skeletally mature patients (53%) in the valgus group, and in one of four patients (25%) in the varus group. Combining antegrade femoral lengthening and temporary HED is a viable option to correct LLD and coronal limb malalignment in skeletally immature patients; however, achieving limb length equalization and realignment may be difficult in cases of severe LLD and angular deformity, in particular.

## 1. Introduction

Leg length discrepancy (LLD) is a common reason for orthopedic consultation. While there is a consensus that differences of less than 2 cm should be treated non-surgically, surgical treatment may be considered for LLD of 2 cm and more [1,2,3]. Leg length equalization can be achieved by reducing the length of the longer leg, either by shortening osteotomy or by epiphysiodesis of the growth plates close to the knee in skeletally immature patients [4,5,6]. However, these procedures reduce final height and may negatively affect body proportions [4,6,7,8]. On the other hand, the lengthening of the shorter leg by distraction osteogenesis with external fixation or intramedullary devices can also be taken into consideration [2,6,9]. While external fixators are associated with adverse effects, such as pin site infection and scarring, intramedullary lengthening nails, in comparison, offer increased patient comfort [9,10,11,12]. Nevertheless, in children, lengthening procedures are commonly performed with external fixators due to open physes and bone dimensions insufficient for the application of intramedullary lengthening devices [9,13]; however, recent studies have shown that antegrade femoral lengthening nails can safely be applied from the age of 8 years onwards [14,15,16,17].

Coronal angular deformities of the knee joint, in particular valgus deformities, are frequently associated with LLD and may further add to their extent [13,18]. Concomitant deformity correction should, thus, be taken into consideration when treating LLD. Combined acute femoral correction osteotomy and subsequent femoral lengthening for the treatment of LLD and coexistent angular deformities reportedly achieves satisfactory results in adults [18,19]. In skeletally immature patients, on the other hand, the realignment of valgus or varus deformities can be achieved by hemiepiphysiodesis (HED) [20,21]. Even though temporary HED close to the knee and femoral distraction osteogenesis using antegrade intramedullary lengthening nails are well-established and widely applied procedures—either in combination or as a standalone treatment—to our knowledge, no studies to date have evaluated their combined application in skeletally immature patients. In the present study, we, hence, investigated the results of the correction of LLD and coronal angular deformities through a combination of proximal femoral distraction osteogenesis using an intramedullary lengthening nail and temporary HED at the distal femur and/or proximal tibia. The primary outcome parameters were pre- and postinterventional leg length, axis realignment and complications associated with treatment, which were analyzed to elucidate whether this combined approach is a suitable treatment regimen in skeletally immature patients.

## 2. Materials and Methods

### 2.1. Study Design and Setting

**Figure 1:**
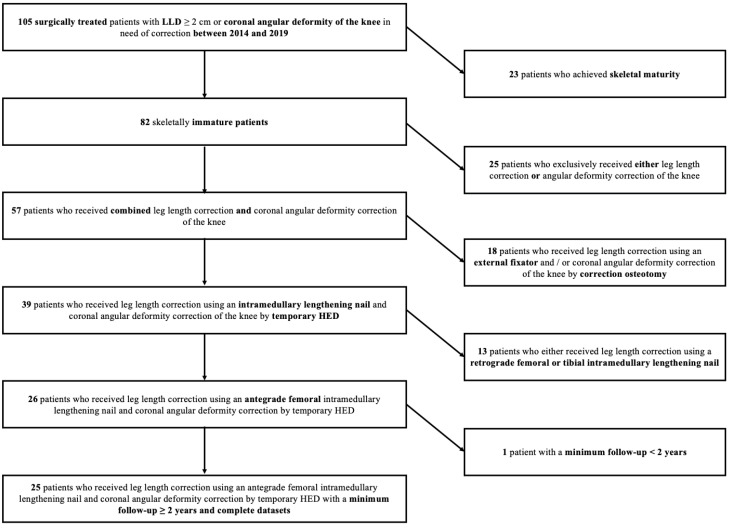
Strengthening the Reporting of Observational studies in Epidemiology (STROBE) diagram detailing the inclusion and exclusion criteria for the study cohort. LLD: Leg length discrepancy. HED: Hemiepiphysiodesis.

In this single-center case series, we identified patients who underwent surgery for correction of LLD and limb malalignment between 2014 and 2019. We retrospectively analyzed the longitudinally maintained database of our tertiary referral university hospital. Inclusion and exclusion criteria are detailed in Figure 1. Twenty-five patients fulfilled the inclusion criteria and were available for analysis. The study findings are reported according to the Strengthening the Reporting of Observational Studies in Epidemiology (STROBE) guidelines [22].

### 2.2. Descriptive Data

The 25 included patients (14 females) were treated for a median initial LLD of 39.0 mm (35.0–45.0). Lengthening procedures were considered in cases of LLD greater than 2 cm, and if the predicted final LLD at skeletal maturity was less than 20 cm. The mean age at implantation of the lengthening nail was 13.4 years (±1.9). A total of 12 of the 25 patients (48%) had received prior lengthening procedures with a median total lengthening of 50.0 mm (47.5–77.5). Underlying medical conditions are shown in Table 1. Patients with simultaneous guided growth and intramedullary lengthening procedures were included in the present study. To improve the comparability of the study results, patients in whom limb lengthening and/or guided growth was performed using other devices (e.g., temporary HED through two-hole plates) were excluded.

Twenty-one patients (84%) presented valgus and four patients (16%) presented varus deformity of the knee. Temporary HED was considered if the mechanical axis deviation (MAD) was greater than 10 mm and the mechanical longitudinal leg axes deviated out of the inner two knee zones (1/−1) [23], and was either conducted prior (*n* = 11), simultaneously (*n* = 10) or subsequently (*n* = 4) to femoral lengthening. The maximum period between HED and lengthening was four years. The median age at initiation of guided growth was 13.2 years (12.3–13.7). Nine of the twenty-five patients (36%) had received temporary HED for correction of limb malalignment prior to the guided growth procedures investigated in the present study. The median initial MAD, mechanical lateral distal femoral angle (mLDFA) and medial proximal tibial angle (MPTA) are shown in Table 2.

The mean follow-up period was 3.7 years (±1.4). The mean age at the time of last follow-up was 16.4 years (±1.9).

### 2.3. Preoperative and Postoperative Clinical and Radiographic Evaluation

Clinical information was acquired through chart review. Anteroposterior long-standing radiographs were obtained from all patients preoperatively and after consolidation. Additionally, full-length standing radiographs were repeated every three months during guided growth. Biplanar radiographs of the femur were taken every second week with the patient under distraction and every six weeks during the consolidation period.

**Figure 2:**
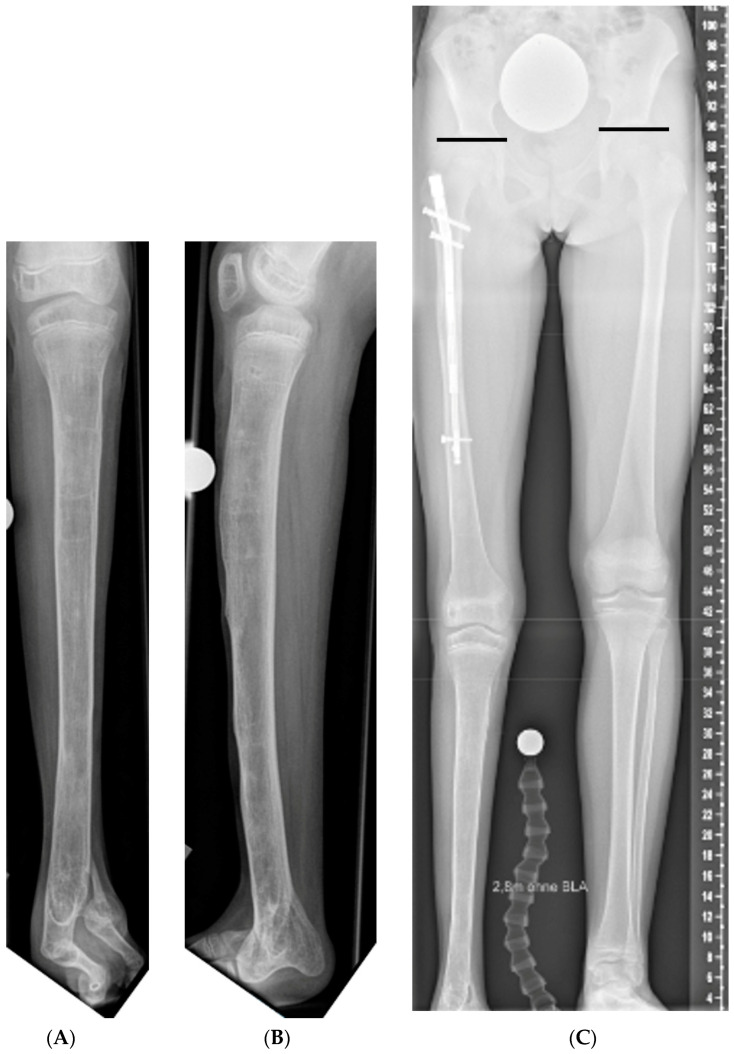
(**A**,**B**) Female patient with preexisting ankle fusion due to fibula hemimelia. (**C**) Residual leg length discrepancy of 1 cm was tolerated to facilitate walking.

The radiological evaluation was performed with regards to the pre- and postinterventional leg length, as well as axis alignment. The aim of treatment was (1) the equalization of leg lengths with a residual LLD of less than 10 mm and (2) limb realignment at skeletal maturity with a MAD of less than 10 mm and within the inner two knee zones (1/−1), as well as joint orientation angles within physiological ranges [23]. Limb alignment and joint orientation were assessed in regard to established parameters [24]. However, in four patients, in whom the predicted final LLD exceeded 70 mm, staged limb lengthening was performed with an initial lengthening of 50 mm. Moreover, in patients with preexisting ankle fusion, undercorrection of 1 cm was tolerated to facilitate gait (Figure 2). In patients with LLD originating from both femur and tibia in whom tibial lengthening was not feasible, up to 4 cm of difference in knee height was accepted [25,26,27]. Patients were counseled on cosmetic aspects and potential abnormalities in gait patterns due to knee height asymmetry prior to treatment.

In guided growth, slight overcorrection of the MAD of 10 mm was intended if skeletal maturity was not expected to be achieved within two years after termination of guided growth.

For deformity analysis and LLD measurements, established techniques known for good interrater reliability were applied as previously described [24]. All measurements were conducted using calibrated radiographs with the PACS^®^ system (GE Healthcare Germany, Solingen, Germany) and the postprocessing software TraumaCad^®^ (Brainlab, Munich, Germany).

### 2.4. Surgical Technique and Perioperative Parameters

All procedures were performed by two senior surgeons (RR, BV), who are also authors of this study.

#### 2.4.1. Limb Lengthening

All femoral lengthening procedures were conducted with the magnetically driven PRECICE^®^ limb lengthening system (NuVasive Specialized Orthopedics, San Diego, CA, USA). A total of 18 of the 25 lengthening nails (72%) were titanium PRECICE^®^ nails and 7 of the 25 nails (28%) were stainless steel PRECICE^®^ Stryde nails. All nails were antegradely inserted via a lateral trochanteric entry [14,15]. The surgical technique was performed as previously described [17].

#### 2.4.2. Guided Growth

Temporary HED was conducted through implantation of FlexTacks^TM^ (Merete GmbH, Berlin, Germany), either at the distal femur with a 30 mm FlexTack^TM^ or at the proximal tibia with a 25 mm FlexTack^TM^. Implantation sites depended on preoperative deformity analysis. Of 21 patients presenting valgus malalignment, 14 received implantation of a flexible staple at the distal medial femur, three received the implantation at the proximal medial tibia, and four received the implantation on both sites concomitantly. Of four patients with varus malalignment, two patients had the staples inserted at the distal lateral femur and two at the proximal lateral tibia, respectively. The implantation was performed according to the manufacturer’s instructions as previously described [21,23,28].

**Figure 3:**
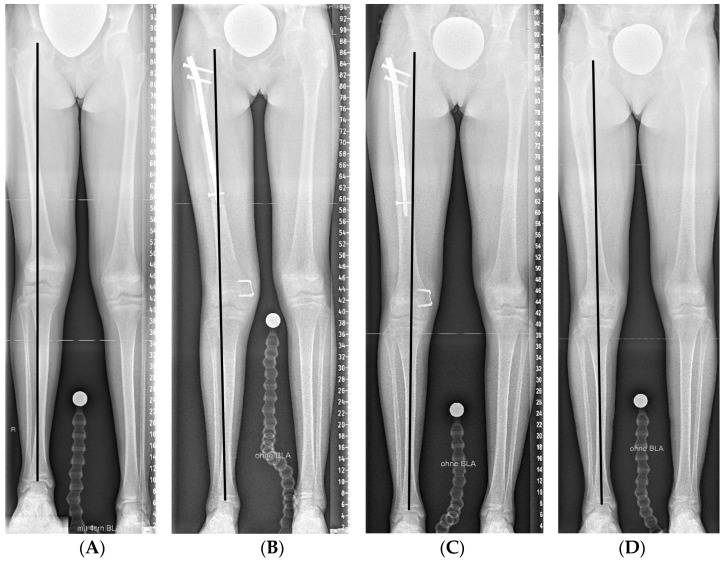
(**A**) Female patient with an idiopathic leg length discrepancy of 37 mm and mild genu valgum. (**B**) Hemiepiphysiodesis and femoral lengthening was conducted simultaneously, with the implantation of the lengthening nail and the flexible staple taking place when the girl was 13 years of age. (**C**,**D**) Implants were retrieved after eight months after sufficient limb realignment and leg length equalization were radiologically observed.

In patients in whom guided growth was performed simultaneously with lengthening, the FlexTack^TM^ implantation was conducted immediately after the lengthening nail had been inserted (Figure 3).

### 2.5. Postoperative Lengthening and Follow-Up Protocol

The postoperative latency period was seven days, and the initial distraction rate was set to 1 mm/day. Patients were allowed partial weight-bearing with 20 kg during distraction. Patients who had received a Stryde nail were allowed full weight-bearing as tolerated. Physiotherapy was recommended at least once per week during lengthening. During distraction, patients were followed every second week in the outpatient clinic. Once the lengthening goal was achieved, a consolidation period of six weeks was initiated. In patients who had received a PRECICE^®^ nail, full weight-bearing was allowed after consolidation of at least three of four cortices was confirmed on bi-planar radiographs.

During guided growth, patients presented at three-month intervals in our outpatient clinic for clinical and radiological follow-up. In patients near the end of skeletal maturation, implants for HED were retrieved as soon as limb realignment was radiologically observed.

### 2.6. Complications

Complications associated with the treatment were summarized descriptively and characterized as minor complications if they resolved without revision surgery and as major complications if they resulted in unplanned revision surgery or permanent functional sequelae [17]. Implant-associated complications, such as breakage and radiological signs of osteolysis, were also assessed [29,30].

### 2.7. Statistical Analysis

SPSS Statistics v28.0.1.0 (IBM, Chicago, IL, USA) was used for all statistical analyses. Data distribution pattern was assessed with the Shapiro–Wilk test. Continuous variables were descriptively analyzed using mean with standard deviation (±) for parametric and median with interquartile range presented as 25th–75th percentile for non-parametric data. Absolute numbers with percentages were given for binary variables.

## 3. Results

### 3.1. Perioperative Parameters

The perioperative parameters are shown in Table 3.

All patients were admitted into the hospital one day prior to their scheduled surgery. After the implantation of flexible staples, a minimum standard length of hospital stay of two days was required. In patients who desired to initiate the lengthening procedure at the hospital, the planned length of hospital stay was ten days to perform radiological verification of distraction at the third day of lengthening.

### 3.2. Implant Retrieval

The removal of lengthening nails was conducted after sufficient consolidation of the regenerate. Flexible staples were retrieved after coronal realignment was achieved. The removal of the lengthening nails was performed at a mean of 16.0 months (±5.6) after implantation, while the mean time from implantation to removal of the flexible staples was 18.9 months (±9.9).

### 3.3. Radiological Assessment

No implant-associated complications, such as breakage or dislocation, were observed. In lengthening procedures performed with a Stryde lengthening nail, focal osteolysis at the junction of the nail was observed in three segments (42%) [31]; however, these findings were exclusively radiological and showed no association with clinical complications, and patients did not report increased pain.

### 3.4. Leg Length Equalization

Radiological data regarding LLD at surgery, predicted LLD at skeletal maturity, LLD after implant removal, and LLD at skeletal maturity are shown in Table 4.

A total of 21 out of 25 patients (84%) showed equalized leg length after the termination of the lengthening procedure. Four patients (16%) showed residual LLD of a mean of 25.3 mm (±14.5). Subsequent lengthening procedures were performed in two patients. At the time of the last follow-up, 13 of the 21 skeletally mature patients (61%) showed equalized leg length, while eight patients presented residual or recurrent LLD of a median of 15.5 mm (12.8–21.8). Two of these patients had preexisting ankle fusion; thus, 1 cm undercorrection of leg length was tolerated. Hence, the lengthening goal was achieved in 15 of the 21 patients (71%).

### 3.5. Axis Realignment

Radiological data regarding the development of MAD, mLDFA and MPTA during and after guided growth are shown in Table 2.

The rebound of valgus malalignment after implant removal was observed in three patients, aged 7, 11 and 13 years, at the time of retrieval of the flexible staple. Two of these patients received subsequent HED. Of 16 patients who had achieved skeletal maturity at the time of last follow-up, nine patients (56%) showed physiological limb alignment. Seven of sixteen patients (44%) showed persistent malalignment with a MAD of more than 10 mm, four of which were valgus deformities, while three patients showed overcorrection towards varus deformity. One patient failed to attend regular follow-up appointments, and, hence, showed varus malalignment at the first follow-up two years after initiation of guided growth, when the patient had already achieved skeletal maturity.

### 3.6. Complications

In total, there were nine complications in eight patients (32%). Of those, three were minor complications occurring in three patients. Six were major complications that required intervention or resulted in permanent functional sequelae, occurring in five patients (Table 5).

In two patients, premature consolidation of the regenerate bone required reosteotomy. Subsequently, distraction was conducted until the lengthening goal was achieved and without further notable events.

**Figure 4:**
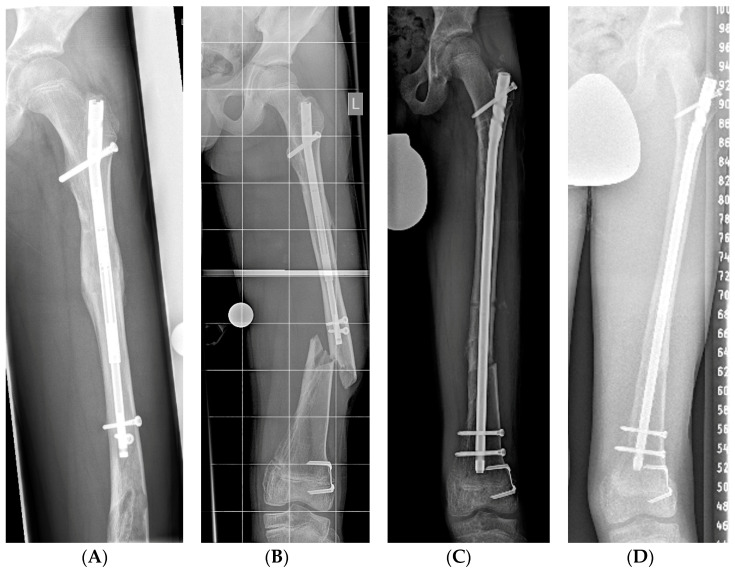
(**A**,**B**) Male patient with Ollier’s disease in whom a pathological fracture occurred at the site of an enchondroma after the lengthening procedure had been completed. (**C**,**D**) Stabilization of the fracture was achieved by exchange nailing.

Two patients suffered a diaphyseal fracture of the femur. One fracture was observed distally to the lengthening nail, 1.5 years after nail implantation. The fracture occurred at the site of an enchondroma in a patient with underlying Ollier’s disease (Figure 4). The second fracture was observed at the site of a screw hole at the distal end of the nail. The patient had performed full weight-bearing against advice. Both fractures occurred after the lengthening procedure had been completed and were successfully treated by exchange nailing with the implantation of a TRIGEN TAN intramedullary nail (Smith + Nephew, Watford, UK).

**Figure 5:**
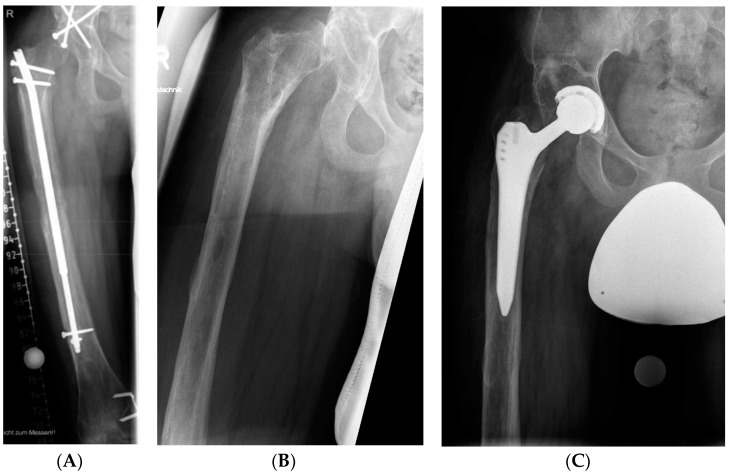
(**A**,**B**) Progressive lateralization of the femoral head during lengthening in a male patient with preexisting congenital femoral deficiency, despite prior attempts to improve hip containment by periacetabular osteotomy. (**C**) The patient ultimately received a total hip arthroplasty due to persistent pain and stiffness of the hip joint.

One hip subluxation was observed after lengthening in a patient with congenital femoral deficiency who had received prior periacetabular osteotomy for the improvement of hip containment and two prior lengthening procedures with a total femoral lengthening of 70 mm (Figure 5). Additionally, the patient suffered an implant-associated infection of the lengthening nail; thus, the nail was removed and a monolateral external fixator (Limb Reconstruction System (LRS), Orthofix, Verona, Italy) was applied until sufficient consolidation of the regenerate bone was observed radiologically. The patient showed persistent restriction and pain in the hip joint and ultimately received a total hip arthroplasty at the age of 18 years. This was the only patient in whom permanent restrictions in joint movement were observed.

There were two temporary knee flexion contractures of 30° that occurred during distraction. In one patient, the distraction rate was temporarily reduced to 0.5 mm/day. In the other patient, the lengthening nail was temporarily retracted until full extension was restored. Lengthening was completed under intensified physiotherapy in both patients without further complications. No permanent functional restrictions of joint movement were observed in these patients.

In one patient, premature consolidation was detected four weeks after surgery. The patient had performed the daily lengthening procedures inconsistently due to fear of pain. Acute distraction of the lengthening nail of 5 mm was performed under sedation. Subsequently, distraction was resumed without further adverse events.

## 4. Discussion

Coronal plane deformities of the knee are frequently encountered in skeletally immature patients presenting LLD, in particular, in the presence of congenital deformities such as fibular hemimelia [32]. Severe coronal malalignment may even lead to the aggravation of preexisting LLD [18]; thus, realignment of the leg should be considered if surgical corrections of LLD are to be undertaken. In this regard, deformity correction and concomitant gradual lengthening through external fixation is a well-established procedure producing reliable results [33]. However, treatment with external fixators is associated with pin tract infections, scarring, and patient discomfort [34,35,36]. These adverse effects may be avoided by application of intramedullary lengthening nails [12,37]. In skeletally mature patients, satisfactory results have been reported for the treatment of LLD and accompanying angular deformities by acute distal femoral deformity correction with plate fixation and successive lengthening with intramedullary lengthening devices [18]. Coronal deformities originating both at the femur and tibia may require an additional correction osteotomy of the tibia [19]. However, in skeletally immature patients, extensive osteotomies can often be avoided by guided growth through temporary HED [21,23]. The realignment of coronal angular deformities may facilitate the use of intramedullary lengthening nails, rather than performing lengthening and deformity correction with an external fixator [38]. Furthermore, in distal femoral deformities concomitant to underlying LLD, the employment of HED around the knee enables intramedullary lengthening at the proximal femur, rather than lengthening at the correction osteotomy site using a retrograde nail; hence, intramedullary lengthening procedures are feasible at an earlier age, since femoral lengthening with an antegrade nail has been shown to be safely applicable in children starting from the age of 8 years [14,15,16]. Femoral lengthening using a retrograde nail, on the other hand, is associated with an increased complication rate [10]. It should preferably be performed in skeletally mature patients to preserve the distal femoral growth plate and avoid premature partial physeal closure and, consequently, recurrent LLD or deformities [13].

Moreover, the application of guided growth allows for performing deformity correction independent of lengthening procedures. Radler et al. reported that lengthening in children and adolescents may be less challenging after deformity correction through guided growth [16]. Furthermore, coronal deformities may occur or exacerbate during lengthening procedures performed along the anatomical axis of the femur due to the discrepancy of approximately 7° between the anatomical and mechanical femoral axis, resulting in a lateral shift of the mechanical axis [39]. Even though recent studies have shown this effect to be of minor importance in lengthening procedures performed in patients with physiologic limb alignment, the potential aggravation of limb malalignment should be taken into consideration when planning lengthening procedures via an antegrade approach in patients with preexisting pathologic valgus malalignment [17,40,41]. In such cases, limb alignment may be regained through temporary HED performed concomitantly or subsequently to lengthening in skeletally immature patients.

We hypothesized that angular coronal deformity correction by temporary HED around the knee, in combination with femoral lengthening with antegrade intramedullary lengthening devices, may offer a promising treatment approach for the combined correction of coronal deformities and LLD in skeletally immature patients, avoiding extensive osteotomies and adverse events associated with lengthening using retrograde nails and external fixation. To our knowledge, this technique has not yet been adequately evaluated in the literature. Even though we observed several complications and a noteworthy amount of patients failed to achieve the desired treatment goals of limb realignment and leg length equalization, a profound correction of the initial deformity was observed in all patients. However, the study findings suggest an increased risk for major complications in patients with underlying conditions associated with alterations of bone and joint morphology. Moreover, we concluded that growth prediction renders difficulty in limbs affected by congenital disorders due to pathological growth patterns, resulting in an increased risk of persistent LLD or insufficient deformity correction at the end of treatment. After HED, deformity rebound may, in particular, occur in cases of implant removal before physeal closure. Even though serial guided growth procedures can be employed for the treatment of residual or recurring malalignment, as long as sufficient growth potential remains, temporary HED should, if feasible, be conducted near skeletal maturity to reduce the risk of persistent limb malalignment [7,21,23].

This study has several limitations. First, this was a retrospective evaluation of a consecutive patient series with a small patient number, with only four individuals included in the varus group. Second, the age at intervention and underlying conditions differed within the study cohort. Interferential statistical analysis was, thus, not feasible. Third, there was no comparison group, and the comparison of results within the patient group was limited due to their heterogeneity. These limitations derived from the fact that underlying conditions and, hence, the indication for this specific treatment are rare. Lastly, not all patients had achieved skeletal maturity at the time of the last follow-up. Long-term results regarding rebound phenomenon and reoccurring LLD will, therefore, have to be assessed at the skeletal maturity of all patients, which may influence the study’s final outcome. However, we believe that the preliminary results of this study are sufficient to draw cautious conclusions on the pitfalls and benefits of the investigated procedure.

## 5. Conclusions

The combination of distal temporary HED and femoral distraction osteogenesis with an antegrade intramedullary lengthening nail appears to be a viable option to correct LLD and coronal angular deformity in skeletally immature patients. A profound correction of the initial deformity was observed in all patients. In cases of severe LLD and limb malalignment, a distinct reduction in these deformities may be associated with considerable improvement of mobility, even if residual deformity persists. It should be emphasized, however, that the indication for this procedure should be cautiously made in patients with underlying comorbidities under careful consideration of their individual risk factors. One should be aware that congenital disorders that cause LLD and limb malalignment are often associated with pathological growth patterns, as well as alterations of bone and joint morphology, which may cause difficulties in achieving the desired treatment goal. Meticulous adherence to a coherent follow-up protocol with regular assessment of limb alignment until skeletal maturity is crucial for the success of treatment. Long-term results, and effects on adjacent joints, in particular, are awaited.

## Figures and Tables

**Table 1:** Underlying conditions.

Etiology	*n*
Fibular hemimelia	9
Congenital femoral deficiency	6
Idiopathic	2
Posttraumatic	2
Hereditary multiple exostoses	2
Enchondromatosis	1
Idiopathic hemihypertrophy	1
Klippel–Trenaunay syndrome	1
Congenital hip dislocation	1

*n*: Number of patients.

**Table 2:** Radiological parameters of guided growth.

	Initial MAD (mm)	MAD after HED (mm)	MAD at Skeletal Maturity (mm)	Initial mLDFA (°)	mLDFA after HED (°)	mLDFA at Skeletal Maturity (°)	Initial MPTA (°)	MPTA after HED (°)	MPTA at Skeletal Maturity (°)
**Valgus deformity**									
*n* = 21	−26.5 (±11.8)	+2.3 (±13.1)		81.8 (±5.3)	89.3 (±4.7)		91.4 (±4.9)	88.6 (±3.2)	
*n* = 17			−7.2 (±15.3)			86.8 (±5.0)			88.9 (±3.0)
**Varus deformity**									
*n* = 4	+30.0 (±16.1)	+2.3 (±14.2)	+15.5 (±11.3)	94.0 (±7.6)	85.0 (±3.4)	88.8 (±2.6)	84.3 (±4.2)	84.8 (±3.4)	84.3 (±3.1)

*n*: Number; mm: millimeter; MAD: mechanical axis deviation; mLDFA: mechanical lateral distal femoral angle; MPTA: medial proximal tibial angle; HED: hemiepiphysiodesis. The parameters are given as mean and standard deviation (±). mLDFA and MPTA of 85–90° were considered physiological [24].

**Table 3:** Perioperative parameters.

	Lengthening Nail Implantation	Flexible Staple Implantation	Combined Implantation
Incision-suture time (minutes)	101.6 (±35.9)	28.5 (26.3–37.5)	156.5 (±62.5)
Hospitalization time (days)	8.1 (±3.8)	2.9 (±1.0)	8.9 (±4.1)

The parameters are given as mean and standard deviation (±) or median and interquartile range presented as 25th–75th percentile.

**Table 4:** Radiological parameters of limb lengthening.

	LLD at Surgery (mm)	Predicted LLD at Skeletal Maturity (mm)	LLD after Implant Retrieval (mm)	LLD after Skeletal Maturity (mm)
*n* = 25	39.0 (35.0–45.0)	44.5 (37.5–58.3)	4.0 (0.0–8.3)	
*n* = 21				10.0 (0.0–12.3)

*n*: Number; mm: millimeter. The parameters are given as median and interquartile range presented as 25th–75th percentile.

**Table 5:** Major complications (revision surgery and/or permanent sequalae) and minor complications (obstacles resolved without revision surgery and without permanent sequalae).

	Number	Type of Complication	Resolved by	Permanent Sequalae
**Major complications** (*n* = 6 in 5 patients)	2	Premature consolidation	Reosteotomy	None
	2	Fracture of the femur	Exchange nailing	None
	1	Deep infection	Retrieval of the lengthening nail, external fixation	Pain, restricted range of movement
	1	Hip subluxation	Total hip arthroplasty	
**Minor complications** (*n* = 3 in 3 patients)	2	Knee flexion contracture	Reduction of distraction rate Temporary retraction of the lengthening nail	None
	1	Premature consolidation	Acute lengthening of 5 mm under sedation	None

*n*: Number.

## Data Availability

The datasets generated and/or analyzed during the current study are available from the corresponding authors on reasonable request.

## References

[1] Gross R.H. (1978). Leg length discrepancy: How much is too much?. Orthopedics.

[2] Vogt B., Gosheger G., Wirth T., Horn J., Rodl R. (2020). Leg Length Discrepancy—Treatment Indications and Strategies. Dtsch. Arztebl. Int..

[3] Quinones D., Liu R., Gebhart J.J. (2020). Study Guide—Leg Length Discrepancy (LLD).

[4] Vogt B., Roedl R., Gosheger G., Frommer A., Laufer A., Kleine-Koenig M.T., Theil C., Toporowski G. (2021). Growth arrest: Leg length correction through temporary epiphysiodesis with a novel rigid staple (RigidTack). Bone Jt. J..

[5] Willegger M., Schreiner M., Kolb A., Windhager R., Chiari C. (2021). Epiphysiodesis for the treatment of tall stature and leg length discrepancy. Wien. Med. Wochenschr..

[6] Hasler C.C. (2000). Leg length inequality. Indications for treatment and importance of shortening procedures. Orthopade.

[7] Vogt B., Schiedel F., Rodl R. (2014). Guided growth in children and adolescents. Correction of leg length discrepancies and leg axis deformities. Orthopade.

[8] Paley D., Bhave A., Herzenberg J.E., Bowen J.R. (2000). Multiplier method for predicting limb-length discrepancy. J. Bone Jt. Surg. Am..

[9] Hafez M., Nicolaou N., Offiah A., Giles S.N., Madan S.S., Fernandes J.A. (2022). Femoral Lengthening in Children-A Comparison Between Magnetic Intramedullary Lengthening Nails and External Fixators. J. Pediatr. Orthop..

[10] Calder P.R., McKay J.E., Timms A.J., Roskrow T., Fugazzotto S., Edel P., Goodier W.D. (2019). Femoral lengthening using the Precice intramedullary limb-lengthening system: Outcome comparison following antegrade and retrograde nails. Bone Jt. J..

[11] Laubscher M., Mitchell C., Timms A., Goodier D., Calder P. (2016). Outcomes following femoral lengthening: An initial comparison of the Precice intramedullary lengthening nail and the LRS external fixator monorail system. Bone Jt. J..

[12] Landge V., Shabtai L., Gesheff M., Specht S.C., Herzenberg J.E. (2015). Patient Satisfaction After Limb Lengthening with Internal and External Devices. J. Surg. Orthop. Adv..

[13] Iobst C.A., Rozbruch S.R., Nelson S., Fragomen A. (2018). Simultaneous Acute Femoral Deformity Correction and Gradual Limb Lengthening Using a Retrograde Femoral Nail: Technique and Clinical Results. J. Am. Acad. Orthop. Surg..

[14] Hammouda A.I., Jauregui J.J., Gesheff M.G., Standard S.C., Herzenberg J.E. (2017). Trochanteric Entry for Femoral Lengthening Nails in Children: Is It Safe?. J. Pediatr. Orthop..

[15] Frommer A., Rodl R., Gosheger G., Vogt B. (2018). Application of motorized intramedullary lengthening nails in skeletally immature patients: Indications and limitations. Unfallchirurg.

[16] Radler C., Mindler G.T., Stauffer A., Weiss C., Ganger R. (2022). Limb Lengthening with Precice Intramedullary Lengthening Nails in Children and Adolescents. J. Pediatr. Orthop..

[17] Frommer A., Roedl R., Gosheger G., Niemann M., Turkowski D., Toporowski G., Theil C., Laufer A., Vogt B. (2021). What Are the Potential Benefits and Risks of Using Magnetically Driven Antegrade Intramedullary Lengthening Nails for Femoral Lengthening to Treat Leg Length Discrepancy?. Clin. Orthop. Relat. Res..

[18] Jardaly A., Gilbert S.R. (2021). Combined antegrade femur lengthening and distal deformity correction: A case series. J. Orthop. Surg. Res..

[19] Steiger C.N., Lenze U., Krieg A.H. (2018). A new technique for correction of leg length discrepancies in combination with complex axis deformities of the lower limb using a lengthening nail and a locking plate. J. Child. Orthop..

[20] Stevens P.M. (2007). Guided growth for angular correction: A preliminary series using a tension band plate. J. Pediatr. Orthop..

[21] Vogt B., Frommer A., Gosheger G., Toporowski G., Tretow H., Rodl R., Laufer A. (2021). Growth modulation through hemiepiphysiodesis: Novel surgical techniques: Risks and progress. Orthopade.

[22] von Elm E., Altman D.G., Egger M., Pocock S.J., Gotzsche P.C., Vandenbroucke J.P., Initiative S. (2007). The Strengthening the Reporting of Observational Studies in Epidemiology (STROBE) statement: Guidelines for reporting observational studies. Epidemiology.

[23] Vogt B., Toporowski G., Gosheger G., Laufer A., Frommer A., Kleine-Koenig M.T., Roedl R., Antfang C. (2023). Guided growth: Angular deformity correction through temporary hemiepiphysiodesis with a flexible staple (FlexTack). Bone Jt. J..

[24] Paley D. (2002). Normal Lower Limb Alignment and Joint Orientation. Principles of Deformity Correction.

[25] Veilleux L.N., AlOtaibi M., Dahan-Oliel N., Hamdy R.C. (2018). Incidence of knee height asymmetry in a paediatric population of corrected leg length discrepancy: A retrospective chart review study. Int. Orthop..

[26] Escott B.G., Kelley S.P. (2012). Management of traumatic physeal growth arrest. Orthop. Trauma.

[27] Stanitski D.F. (1999). Limb-length inequality: Assessment and treatment options. J. Am. Acad. Orthop. Surg..

[28] Sattelberger J., Hillebrand H., Gosheger G., Laufer A., Frommer A., Appelbaum S., Abood A.A., Gottliebsen M., Rahbek O., Moller-Madsen B. (2021). Comparison of histomorphometric and radiographic effects of growth guidance with tension-band devices (eight-Plate and FlexTack) in a pig model. Acta Orthop..

[29] Rolfing J.D., Bunger M., Petruskevicius J., Abood A.A. (2021). Removal of broken precice stryde intramedullary lengthening nails. Orthop. Traumatol. Surg. Res..

[30] Iobst C.A., Frost M.W., Rolfing J.D., Rahbek O., Bafor A., Duncan M., Kold S. (2021). Radiographs of 366 removed limb-lengthening nails reveal differences in bone abnormalities between different nail types. Bone Jt. J..

[31] Jellesen M.S., Lomholt T.N., Hansen R.Q., Mathiesen T., Gundlach C., Kold S., Nygaard T., Mikuzis M., Olesen U.K., Rolfing J.D. (2021). The STRYDE limb lengthening nail is susceptible to mechanically assisted crevice corrosion: An analysis of 23 retrieved implants. Acta Orthop..

[32] Paley D. (2016). Surgical reconstruction for fibular hemimelia. J. Child. Orthop..

[33] Horn J., Steen H., Huhnstock S., Hvid I., Gunderson R.B. (2017). Limb lengthening and deformity correction of congenital and acquired deformities in children using the Taylor Spatial Frame. Acta Orthop..

[34] Tsuchiya H., Uehara K., Abdel-Wanis M.E., Sakurakichi K., Kabata T., Tomita K. (2002). Deformity correction followed by lengthening with the Ilizarov method. Clin. Orthop. Relat. Res..

[35] Iobst C., Liu R. (2016). A systematic review of incidence of pin track infections associated with external fixation. J. Limb Lengthening Reconstr..

[36] Bue M., Bjarnason A.O., Rolfing J.D., Larsen K., Petruskevicius J. (2021). Prospective evaluation of pin site infections in 39 patients treated with external ring fixation. J. Bone Jt. Infect..

[37] Hafez M., Nicolaou N., Offiah A., Offorha B., Giles S., Madan S., Fernandes J.A. (2022). Quality of life of children during distraction osteogenesis: A comparison between intramedullary magnetic lengthening nails and external fixators. Int. Orthop..

[38] Stevens P.M. (2016). The role of guided growth as it relates to limb lengthening. J. Child. Orthop..

[39] Burghardt R.D., Paley D., Specht S.C., Herzenberg J.E. (2012). The effect on mechanical axis deviation of femoral lengthening with an intramedullary telescopic nail. J. Bone Jt. Surg. Br..

[40] Wagner P., Burghardt R.D., Green S.A., Specht S.C., Standard S.C., Herzenberg J.E. (2017). PRECICE((R)) magnetically-driven, telescopic, intramedullary lengthening nail: Pre-clinical testing and first 30 patients. SICOT J..

[41] Baumgart R. (2009). The reverse planning method for lengthening of the lower limb using a straight intramedullary nail with or without deformity correction. A new method. Oper. Orthop. Traumatol..

